# Sociodemographic determinants and clinical risk factors associated with COVID-19 severity: *a cross-sectional analysis of over 200,000 patients in Tehran, Iran*

**DOI:** 10.1186/s12879-021-06179-4

**Published:** 2021-05-25

**Authors:** Mohammad-Reza Sohrabi, Rozhin Amin, Ali Maher, Ayad Bahadorimonfared, Shahriar Janbazi, Khatereh Hannani, Ali-Asghar Kolahi, Ali-Reza Zali

**Affiliations:** 1 Community Medicine Department, School of Medicine, Shahid Beheshti University of Medical Sciences, Tehran, Iran; 2 Social Determinants of Health Research Center, Shahid Beheshti University of Medical Sciences, Tehran, Iran; 3 School of Management and Medical Education, Shahid Beheshti University of Medical Sciences, Tehran, Iran; 4 Statistics & Information Technology Management, Shahid Beheshti University of Medical Sciences, Tehran, Iran; 5 Functional Neurosurgery Research Center, Shahid Beheshti University of Medical Sciences, Tehran, Iran

**Keywords:** Blood oxygen saturation, COVID-19, Determinants, Health outcome, Iran, Severity, SARS-CoV-2

## Abstract

**Background:**

Defining socio-demographic factors, clinical presentations and underlying diseases associated with COVID-19 severity could be helpful in its management. This study aimed to further clarify the determinants and clinical risk factors of the disease severity in patients infected with COVID-19.

**Methods:**

A multi-centre descriptive study on all patients who have been diagnosed with COVID-19 in the province of Tehran from March 2020 up to Dec 2020 was conducted. Data on socio-demographic characteristics, clinical presentations, comorbidities, and the health outcomes of 205,654 patients were examined. Characteristics of the study population were described. To assess the association of study variables with the disease severity, the Chi-Squared test and Multiple Logistic Regression model were applied.

**Results:**

The mean age of the study population was 52.8 years and 93,612 (45.5%) were women. About half of the patients have presented with low levels of blood oxygen saturation. The ICU admission rate was 17.8% and the overall mortality rate was 10.0%. Older age, male sex, comorbidities including hypertension, cancer, chronic respiratory diseases other than asthma, chronic liver diseases, chronic kidney diseases, chronic neurological disorders, and HIV/AIDS infection were risk markers of poor health outcome. Clinical presentations related with worse prognosis included fever, difficulty breathing, impaired consciousness, and cutaneous manifestations.

**Conclusion:**

These results might alert physicians to pay attention to determinants and risk factors associated with poor prognosis in patients with COVID-19. In addition, our findings aid decision makers to emphasise on vulnerable groups in the public health strategies that aim at preventing the spread of the disease and its mortalities.

## Background

The Severe Acute Respiratory Syndrome Coronavirus 2 (SARS-CoV-2) has now affected every corner of the world. The virus was first identified in China in the late 2019, leading to a wide variety of events including flu-like, gastrointestinal, and neurological symptoms. The disease was named as COVID-19 and was stated as a “Public Health Emergency of International Concern” by the World Health Organization (WHO) in early 2020 [1, 2]. Within a few months the illness spread to more than 200 countries around the globe with about 70 million confirmed cases and over a million and a half confirmed deaths to date (December 10, 2020) [3].

*Iran* is one of the first and worst affected countries by SARS-CoV-2 virus, worldwide. The outbreak started from the province of Qom, where two confirmed deaths due to COVID-19 were officially reported on February 19. Shortly after, in early March, the disease reached nearly every province throughout the country [4]. Since then, massive public health interventions have been imposed across the country to contain the spread of the disease. The pubic health measures included adopting face mask and physical distancing in enclosed public places, imposing inter- and intra- city commuting restrictions, prohibiting indoor events, and limiting social gatherings. Though the suppression strategies have been successful in reducing the cases, counts appear to resurge easily when these control measures are relaxed. As of December 9th 2020, the country has experienced three major peaks in the COVID-19 epidemic curve, leading to about 1,100,000 COVID-19 confirmed cases, and 52,000 confirmed mortalities [5].

As the pandemic wears on, the health and socio-economic consequences continue to grow. Describing the clinical features and associated outcomes of patients diagnosed with coronavirus disease is crucial in improving our understanding about the disease, in optimising resource allocation to patients with the highest risk of severe outcome, and in effectively managing the pandemic. Several articles have been published worldwide, defining the characteristics and outcomes of different cohorts of patients with COVID-19 [6–11]. However, most studies are single centre and are focused on hospitalized patients. Therefore, more research is needed to support evidence informed decision making and to enhance public awareness. This paper was intended to provide a more holistic view of the disease by using real-time data from a large multi centre group of patients diagnosed with COVID-19, including patients with mild symptoms. The aim of the study was to summarize the socio-demographic and clinical characteristics of the 205,654 patients who were diagnosed with COVID-19 in Tehran, and to identify the predictors of severe health outcomes.

## Methods

A descriptive epidemiological study was conducted using the registry database of Coronavirus Control Operations Headquarter in the province of Tehran. The province is located in the north-central region of Iran, and is home to the country’s capital city of Tehran. The most recent national population census held in 2016, counted a total population of 13,267,637 for the province, with 8,693,706 (equivalent to 70% of the inhabitants) living in urban areas. It is the country’s most populous and important COVID-19 epicentre [12].

In Iran, the national registry for novel coronavirus disease was established in March 2020. All suspected, probable, and confirmed case data were prospectively recorded on the national registry of COVID-19 database, by using WHO case definition guidance [13]. In this multi-centre study, all COVID-19 cases who had visited COVID-19 designated healthcare facilities across the province of Tehran from March 2020 up to Dec 2020 were included. Data on socio-demographic characteristics, clinical presentations, comorbidities, and the health outcomes of 205,654 patients were examined. Of note, all healthcare facilities involved in the treatment of patients with COVID-19 related symptoms were officially designated by the Ministry of Health and Medical education (MOHME) after the outbreak was declared in early March 2020.

### Variables

Patients’ data included age, sex, residing area, smoking history, opioids history, history of exposure to SARS-CoV-2, Clinical presentations (fever, cough, muscle ache, difficulty breathing, chest pain, loss of smell, loss of taste, loss of appetite, nausea, diarrhea, headache, vertigo, impaired consciousness, seizure, paresis, paraplegia, skin lesions), comorbidities (diabetes, hypertension, cardiovascular diseases (CVD), cancer, asthma, chronic respiratory diseases other than asthma, chronic liver diseases, chronic kidney diseases, chronic neurological diseases, chronic haematological diseases, HIV/AIDS, chronic immune deficiency diseases other than HIV/AIDS), blood oxygen saturation (SpO2%), chest CT findings, intensive care unit (ICU) admission, and the disease outcome.

All variables were categorized for ease of interpretation. Patients were split into 9 age groups: 0–9, 10–19, 20–29, 30–39, 40–49, 50–59, 60–79, and 80 years and above. Sex was defined as being a woman or a man. Residing area was divided into 3 regions based on regional boundaries of three public health networks who were responsible for administration of health services in the province of Tehran. The categories were north and east, centre and south, and west. The blood oxygen saturation was classified based on the National Coronavirus Control Operations Headquarter protocol as being either higher than 93%, or 93% and lower. The disease outcome was categorized as survived, and deceased. All other variables were documented as yes or no. The history of exposure to SARS-CoV-2 was defined as having any recent contacts with a probable or confirmed case of COVID-19 from 2 days prior to the onset of the symptoms till 14 days post symptom onset, and was assessed by questioning the patient about the contact.

### Statistical analysis

Descriptive statistics (absolute number and percentage) were used to show the characteristics of the study population. The Chi-Squared test was performed to analyse the association of the patients’ characteristics with the blood oxygen saturation level and disease outcome. Risk factors associated with low blood oxygen saturation levels and death were explored by fitting the multivariate logistic regression model to the data. All estimates were examined by using IBM SPSS Statistics, version 26 (IBM Corp., Armonk, N.Y., USA), and the significance level was set at α < 0.05.

The data was collected by trained health care professionals and based on a standardized reporting form, therefore the percentages of missing values were low and ranged between 1 to 5% across all variables used for this study. Hence, the effect of the missing data on the validity of statistical inferences was regarded as insignificant [14].

The authors confirm that all methods were carried out in accordance with relevant guidelines and regulations, including the Declaration of Helsinki.

## Results

In this paper, data on 205,654 patients who were diagnosed with COVID-19 in the province of Tehran from March 2020 up to Dec 2020 were analysed. Of the study population, 64,468 (31.3%) were confirmed cases as defined by having positive SARS-CoV-2 PCR test result; the remaining were diagnosed according to COVID-19 associated changes in chest CT and clinically-epidemiologically criteria. Chest CT was performed for 121,973 patients, of whom 91% had shown COVID-19 associated changes. The mean ± SD age of the analytic population was 52.8 ± 21.1 years (Median 54.0 years) and 93,612 (45.5%) were women. About half of the patients (48.2%, *n* = 99,161) have presented with low levels of blood oxygen saturation (mean ± SD age: 58.6 ± 19.86). The ICU admission rate was 17.8% (*n* = 36,692) with the mean age of 60.9 ± 20.98 for patients who required ICU admission. Overall, 20,472 individuals (10.0%) have died with the disease and the mean age of deceased patients was 67.4 ± 17.37.

Patients with 60 to 69 years of age represented the most common age group in the study population. About 25% aged over 70 years, and 6% (*n* = 2857) were among the children under the age of 19. The majority resided in the north and east regions of the province. Hypertension, diabetes, and cardiovascular diseases were the most common reported comorbidities. The most common symptom on admission was cough, followed by difficulty breathing, fever, and muscle ache. With regard to gastrointestinal manifestations, nausea was the most common complaint. Skin lesions and paraplegia were relatively rare conditions (Table 1).

**Table 1:** Characteristics and clinical presentations in patients with COVID-19

Characteristics	Women		Men		Overall	
	n	%	n	%	n	%
Age (years)						
0–9	3920	4.2	4955	4.4	8875	4.3
10–19	1627	1.7	1931	1.7	3558	1.7
20–29	6278	6.7	7417	6.6	13695	6.7
30–39	13345	14.3	16332	14.6	29677	14.4
40–49	13502	14.4	16773	15.0	30275	14.7
50–59	15551	16.6	18758	16.7	34309	16.7
60–69	16471	17.6	19338	17.3	35809	17.4
70–79	13189	14.1	14753	13.2	27942	13.6
80 and over	9729	10.4	11785	10.5	21514	10.5
Residing area						
West	32287	34.5	37607	33.6	69894	34.0
Central, South	23137	24.7	26167	23.4	49304	24.0
North, East	38188	40.8	48268	43.1	86456	42.0
Positive history of smoking	421	0.4	2735	2.4	3156	1.5
Positive history of opioids	306	0.3	1595	1.4	1901	0.9
Positive history of exposure to SARS-CoV-2	30920	33.0	37497	33.5	68417	33.3
Underlying diseases						
Diabetes	10384	11.1	9791	8.7	20175	9.8
Hypertension	10827	11.6	10019	8.9	20846	10.1
CVD	8798	9.4	10558	9.4	19355	9.4
Cancer	1906	2.0	2163	1.9	4069	2.0
Asthma	1488	1.6	1246	1.1	2734	1.3
Chronic respiratory diseases	1375	1.5	1781	1.6	3156	1.5
Chronic liver diseases	429	0.5	535	0.5	964	0.5
Chronic kidney diseases	1776	1.9	2477	2.2	4253	2.1
Chronic neurological diseases	896	1.0	991	0.9	1887	0.9
Chronic immune deficiency diseases	364	0.4	262	0.2	626	0.3
Chronic haematological diseases	520	0.6	493	0.4	1013	0.5
HIV/AIDS	73	0.1	81	0.1	154	0.1
Clinical presentations						
Fever	31914	34.1	41465	37.0	73379	35.7
Cough	43317	46.3	52321	46.7	95638	46.5
Muscle ache	28439	30.4	32655	29.1	61094	29.7
Difficulty breathing	39842	42.6	49102	43.8	88944	43.2
Chest pain	2476	2.6	2833	2.5	5309	2.6
Loss of smell	1909	2.0	2055	1.8	3964	1.9
Loss of taste	1189	1.3	1309	1.2	2498	1.2
Loss of appetite	4623	4.9	5491	4.9	10114	4.9
Nausea	5243	5.6	4860	4.3	10103	4.9
Diarrhea	2969	3.2	3371	3.0	6340	3.1
Headache	5589	6.0	5558	5.0	11147	5.4
Vertigo	1874	2.0	1922	1.7	3796	1.8
Impaired consciousness	3522	3.8	4554	4.1	8076	3.9
Seizure	311	0.3	366	0.3	677	0.3
Paresis	348	0.4	452	0.4	800	0.4
Paraplegia	127	0.1	129	0.1	256	0.1
Skin lesions	97	0.1	104	0.1	201	0.1
Positive chest CT findings	49475	52.9	62371	55.7	111846	54.4
Admitted to ICU	15899	17.0	20793	18.6	36692	17.8

*n* number of patients in each sub-category, *%* percentage from the total number of patients

**Fig. 1:**
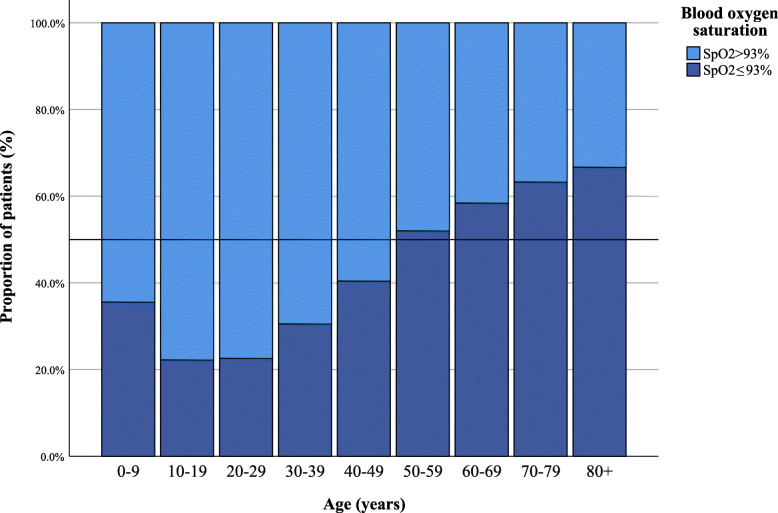
Blood oxygen saturation level by age group in patients with COVID-19

Patients aged 80 and over, showed the highest proportion of patients experiencing low levels of blood oxygen saturation. Lowest rate was observed in patients between the ages of 10 to 19 (Fig. 1). More men patients had SpO2 of less than 94% compared to women. Greater proportion of patients have presented with low blood oxygen saturation level in north and east public health region, compared to the other two public health areas in the province. Having a positive history for opioids was significantly associated with lower levels of SpO2. However, the association was insignificant for patients with positive history of smoking. Comorbidities including hypertension, diabetes, cardiovascular diseases, asthma, chronic respiratory diseases, chronic kidney diseases, and chronic neurological disorders were associated with lower SpO2 in COVID-19 patients. The relation between low SpO2 and Chronic liver diseases, chronic immune deficiency disorders, chronic haematological diseases, and HIV/AIDS was not significant. Among the symptoms, all clinical presentations were correlated with low levels of blood oxygen saturation, except for nausea, vertigo, paraplegia, and skin lesions (Table 2).

**Table 2:** The distribution of blood oxygen saturation level and disease outcome in patients with COVID-19

Characteristics	SpO2 ≤ 93%		SpO2 > 93%		*P* value	Deceased		Survived		*P* value
	n	%	n	%		n	%	n	%	
Age (years)										
0–9	3158	3.2	5717	5.4	< 0.001	311	1.5	8564	4.6	< 0.001
10–19	791	0.8	2767	2.6		110	0.5	3448	1.9	
20–29	3094	3.1	10601	10.0		234	1.1	13461	7.3	
30–39	9067	9.1	20610	19.4		695	3.4	28982	15.7	
40–49	12241	12.3	18034	16.9		1342	6.6	28933	15.6	
50–59	17845	18.0	16464	15.5		2625	12.8	31684	17.1	
60–69	20928	21.1	14881	14.0		4622	22.6	31187	16.8	
70–79	17684	17.8	10258	9.6		5110	25.0	22832	12.3	
80 and over	14353	7.0	7161	3.5		5425	26.5	16089	8.7	
Sex										
Women	43053	43.4	50559	47.5	< 0.001	8326	40.7	85286	46.1	< 0.001
Men	56108	56.6	55934	52.5		12148	59.3	99894	53.9	
Residing area										
West	32089	32.4	37805	35.5	< 0.001	6159	30.1	63735	34.4	< 0.001
Central, South	19738	19.9	29566	27.8		4707	23.0	44597	24.1	
North, East	47334	47.7	39122	36.7		9608	46.9	76848	41.5	
Positive history of smoking	1522	1.5	1634	1.5	0.99	316	1.5	2840	1.5	0.91
Positive history of opioids	1075	1.1	826	0.8	< 0.001	275	1.3	1626	0.9	< 0.001
Positive history of exposure to SARS-CoV-2	39671	40.0	28746	27.0	< 0.001	7154	34.9	61263	33.1	< 0.001
Underlying diseases										
Diabetes	12012	12.1	8163	7.7	< 0.001	3188	15.6	16987	9.2	< 0.001
Hypertension	13164	13.3	7682	7.2	< 0.001	3241	15.8	17605	9.5	< 0.001
CVD	10960	11.1	8395	7.9	< 0.001	3148	15.4	16207	8.8	< 0.001
Cancer	2133	2.2	1936	1.8	< 0.001	786	3.8	3283	1.8	< 0.001
Asthma	1590	1.6	1144	1.1	< 0.001	271	1.3	2463	1.3	0.93
Chronic respiratory diseases	2023	2.0	1133	1.1	< 0.001	556	2.7	2600	1.4	< 0.001
Chronic liver diseases	466	0.5	498	0.5	0.93	141	0.7	823	0.4	< 0.001
Chronic kidney diseases	2488	2.5	1765	1.7	< 0.001	920	4.5	3333	1.8	< 0.001
Chronic neurological diseases	1120	1.1	767	0.7	< 0.001	351	1.7	1536	0.8	< 0.001
Chronic immune deficiency diseases	303	0.3	323	0.3	0.92	68	0.3	558	0.3	0.44
Chronic haematological diseases	505	0.5	508	0.5	0.29	158	0.8	855	0.5	< 0.001
HIV/AIDS	86	0.1	68	0.1	0.05	32	0.2	122	0.1	< 0.001
Clinical presentations										
Fever	37864	38.2	35515	33.3	< 0.001	7134	34.8	66245	35.8	0.008
Cough	45873	46.3	49765	46.7	0.03	7943	38.8	87695	47.4	< 0.001
Muscle ache	27006	27.2	34088	32.0	< 0.001	4700	23.0	56394	30.5	< 0.001
Difficulty breathing	55060	55.5	33884	31.8	< 0.001	12839	62.7	76105	41.1	< 0.001
Chest pain	2692	2.7	2617	2.5	< 0.001	566	2.8	4743	2.6	0.08
Loss of smell	1730	1.7	2234	2.1	< 0.001	250	1.2	3714	2.0	< 0.001
Loss of taste	1149	1.2	1349	1.3	0.02	169	0.8	2329	1.3	< 0.001
Loss of appetite	5476	5.5	4638	4.4	< 0.001	1110	5.4	9004	4.9	< 0.001
Nausea	4915	5.0	5188	4.9	0.37	793	3.9	9310	5.0	< 0.001
Diarrhea	2621	2.6	3719	3.5	< 0.001	424	2.1	5916	3.2	< 0.001
Headache	4940	5.0	6207	5.8	< 0.001	691	3.4	10456	5.6	< 0.001
Vertigo	1839	1.9	1957	1.8	0.77	356	1.7	3440	1.9	0.23
Impaired consciousness	6015	6.1	2061	1.9	< 0.001	3213	15.7	4863	2.6	< 0.001
Seizure	262	0.3	415	0.4	< 0.001	67	0.3	610	0.3	0.95
Paresis	429	0.4	371	0.3	0.002	132	0.6	668	0.4	< 0.001
Paraplegia	143	0.1	113	0.1	0.01	44	0.2	212	0.1	< 0.001
Skin lesions	89	0.1	112	0.1	0.26	16	0.1	185	0.1	0.34
Positive chest CT findings	66027	66.6	45819	43.0	< 0.001	13408	65.5	98438	53.2	< 0.001
Admitted to ICU	23912	24.1	12780	12.0	< 0.001	11011	53.8	25681	13.9	< 0.001

*n* number of patients in each sub-category, *%* percentage of patients in each sub-category; *P*-value obtained from Pearson Chi-Square test

**Fig. 2:**
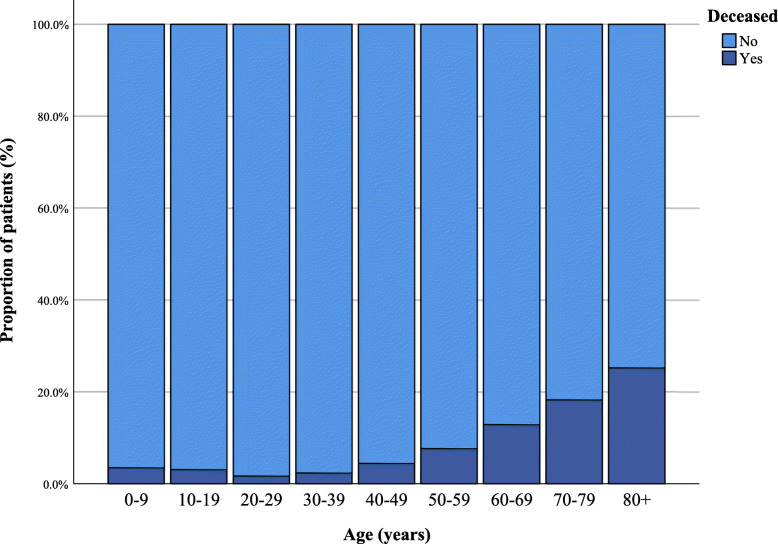
Disease outcome by age group in patients with COVID-19

In this study, one in every 10 patients died because of COVID-19. The highest death rate was obtained in people aged 80 and above, and the lowest rate in children with 10 to 19 years of age, developing a J shaped curve for the age distribution of mortality by COVID-19 (Fig. 2). COVID-19 mortality was higher in men, and in the public health territories of the north and east region. Death rate was significantly higher in COVID-19 patients who had suffered from comorbidities including diabetes, hypertension, cardiovascular diseases, cancer, chronic respiratory diseases, chronic liver diseases, chronic kidney diseases, and chronic neurological disorders, chronic haematological diseases, and HIV/AIDS. Asthma, and chronic immune deficiency disorders were not associated with higher mortality. All COVID-19 related symptoms were significantly correlated with higher mortality, except for chest pain, vertigo, seizure, and skin lesions (Table 2).

Results from the multivariate logistic regression analysis of the predictors of COVID-19 related hypoxia are provided in Table 3. Population aged 60 years and older had increased odds of death due to COVID-19. Male patients were more likely to experience low blood oxygen saturation levels, compared to female patients. People residing in public health region of north and east, showed a significantly increased risk of low SpO2 levels when they were infected with SARS-CoV-2. After accounting for other covariates, positive history of smoking revealed a protective effect against hypoxia in COVID-19 infected patients. Yet, the association between opioids dependency and low levels of blood oxygen in SARS-CoV-2 infected patients remained significant. In the multivariate analysis, patients with underlying conditions containing diabetes, hypertension, cardiovascular diseases, chronic respiratory diseases, and chronic kidney diseases were more likely to present with low SpO2 saturation levels related to COVID-19 infection. Remaining comorbidities displayed insignificant associations. Clinical presentations including fever, muscle ache, difficulty breathing, diarrhea, headache, impaired consciousness, and skin lesions were associated with greater likelihood of developing low oxygen levels from COVID-19.

**Table 3:** Logistic regression model of independent variables associated with SpO2 levels in patients with COVID-19

Variables	aOR	95% Confidence Interval		*P* value
		Lower	Upper	
Age (years)				
0–19	1			
20–39	0.52	0.492	0.563	< 0.001
40–59	0.94	0.889	1.010	0.09
60 and over	1.51	1.422	1.616	< 0.001
Sex				
Women	1			
Men	1.15	1.124	1.181	< 0.001
Residing area				
West	1			
Central, South	1.15	1.11	1.20	< 0.001
North, East	1.66	1.622	1.712	< 0.001
Positive history of smoking	0.82	0.747	0.904	< 0.001
Positive history of opioids	1.15	1.027	1.306	0.01
Positive history of exposure to SARS-CoV-2	1.76	1.724	1.815	< 0.001
Positive chest CT findings	3.10	2.956	3.256	< 0.001
Underlying diseases				
Diabetes	0.92	0.890	0.968	< 0.001
Hypertension	1.12	1.083	1.174	< 0.001
CVD	0.74	0.717	0.780	< 0.001
Cancer	0.916	0.842	0.997	0.43
Asthma	1.03	0.933	1.154	0.49
Chronic respiratory diseases	1.26	1.147	1.398	< 0.001
Chronic liver diseases	0.86	0.726	1.030	0.10
Chronic kidney diseases	0.89	0.828	0.976	0.01
Chronic neurological diseases	1.12	0.989	1.271	0.07
Chronic immune deficiency diseases	1.08	0.868	1.359	0.47
Chronic haematological diseases	0.85	0.722	1.020	0.82
HIV/AIDS	1.34	0.865	2.096	0.18
Clinical presentations				
Fever	1.40	1.370	1.444	< 0.001
Cough	0.99	0.970	1.021	0.70
Muscle ache	0.88	0.861	0.910	< 0.001
Difficulty breathing	2.43	2.369	2.493	< 0.001
Chest pain	0.99	0.926	1.067	0.87
Loss of smell	1.00	0.915	1.107	0.89
Loss of taste	1.07	0.949	1.215	0.26
Loss of appetite	0.99	0.940	1.045	0.73
Nausea	1.02	0.967	1.080	0.44
Diarrhea	0.81	0.764	0.879	< 0.001
Headache	0.84	0.802	0.891	< 0.001
Vertigo	0.95	0.880	1.042	0.31
Impaired consciousness	2.26	2.115	2.419	< 0.001
Seizure	1.01	0.812	1.272	0.88
Paresis	1.07	0.907	1.285	0.39
Paraplegia	0.87	0.648	1.192	0.40
Skin lesions	1.57	1.055	2.362	0.02
Constant	0.152			< 0.001

Table 4 demonstrates the multivariate logistic regression analysis of the predictors of COVID-19 related death. The odds of death from COVID-19 increased with age, except for the age group of 20 to 39. Men were more likely to die of the disease compared to women. Living in the public health region of central and south was associated with higher risk of death. Patients with positive history of smoking were less likely to die of COVID-19, than their counterparts. However, the association between the positive history of opioids dependency and COVID-19 death was insignificant in multivariate analysis. Among the comorbidities, cancer, asthma, chronic respiratory diseases, chronic liver diseases, chronic kidney diseases, chronic neurological disorders, and HIV/AIDS were associated with increased risk of death in patients with SARS-CoV-2 infection. Diabetes, hypertension, cardiovascular diseases, chronic immune deficiency disorders, and chronic haematological diseases were insignificantly correlated the COVID-19 mortality rate. Clinical presentations including fever, cough, muscle ache, difficulty breathing, blood oxygen saturation of less than 94%, nausea, headache, and impaired consciousness were associated with higher risk of death in patients infected with SARS-CoV-2.

**Table 4:** Logistic regression model of independent variables associated with COVID-19 mortality

Variables	aOR	95% Confidence Interval		*P* value
		Lower	Upper	
Age (years)				
0–19	1			
20–39	0.74	0.635	0.884	0.001
40–59	1.48	1.275	1.720	< 0.001
60 and over	3.70	3.197	4.283	< 0.001
Sex				
Women	1			
Men	1.23	1.186	1.281	< 0.001
Residing area				
West	1			
Central, South	1.52	1.444	1.615	< 0.001
North, East	1.42	1.367	1.491	< 0.001
History of smoking	0.76	0.659	0.893	0.001
History of opioids	1.09	0.930	1.289	0.27
History of exposure to SARS-CoV-2	0.96	0.923	0.999	0.04
Positive chest CT findings	2.29	2.060	2.565	< 0.001
Underlying diseases				
Diabetes	1.04	0.984	1.102	0.16
Hypertension	0.97	0.923	1.029	0.34
CVD	1.01	0.955	1.071	0.69
Cancer	1.71	1.539	1.905	< 0.001
Asthma	0.76	0.649	0.904	0.002
Chronic respiratory diseases	1.15	1.022	1.315	0.02
Chronic liver diseases	1.41	1.112	1.803	0.005
Chronic kidney diseases	1.75	1.588	1.938	< 0.001
Chronic neurological diseases	1.16	1.001	1.364	0.04
Chronic immune deficiency diseases	1.18	0.828	1.699	0.35
Chronic haematological diseases	1.23	0.976	1.570	0.07
HIV/AIDS	1.77	1.053	2.973	0.03
Symptoms				
Fever	1.09	1.099	1.143	< 0.001
Cough	0.83	0.801	0.868	< 0.001
Muscle ache	0.87	0.845	0.924	< 0.001
Difficulty breathing	1.63	1.568	1.701	< 0.001
SpO2 < 93%	2.67	2.547	2.801	< 0.001
Chest pain	1.08	0.970	1.204	0.16
Loss of smell	0.90	0.760	1.065	0.21
Loss of taste	0.91	0.738	1.124	0.38
Loss of appetite	1.05	0.969	1.137	0.23
Nausea	0.85	0.775	0.934	0.001
Diarrhea	0.89	0.790	1.012	0.07
Headache	0.78	0.711	0.861	< 0.001
Vertigo	0.99	0.864	1.136	0.89
Impaired consciousness	3.73	3.508	3.979	< 0.001
Seizure	0.97	0.699	1.366	0.89
Paresis	1.19	0.949	1.491	0.13
Paraplegia	0.74	0.497	1.123	0.16
Skin lesions	0.85	0.450	1.619	0.62
Constant	0.006			< 0.001

## Discussion

This research is a multicentre descriptive study on the socio-demographic determinants and clinical characteristics of a large group of 205,654 patients with COVID-19. The main findings showed that older age (60 and older), male sex, residing in the north and east region of the province, having a positive history for opioids dependency, and having COVID-19 exposure history were associated with low levels of blood oxygen saturation in patients infected with SARS-CoV-2. Patients suffering from comorbidities including diabetes, hypertension, cardiovascular diseases, chronic respiratory, and chronic kidney diseases were more likely to develop low SpO2 levels. Clinical manifestations correlated with the COVID-19 related hypoxia were fever, muscle ache, difficulty breathing, diarrhea, headache, impaired consciousness, and skin lesions. The positive predictors of mortality due to COVID-19 were older age (60 and older), male sex, residing in the public health region of central and south, and having COVID-19 exposure history. Comorbidities containing cancer, asthma, chronic respiratory diseases, chronic liver and chronic kidney diseases, chronic neurological disorders, and HIV/AIDS infection were associated with higher risk of death in patients infected with SARS-CoV-2. Fever, cough, muscle ache, difficulty breathing, SpO2 ≤ 93, nausea, headache, and impaired consciousness were clinical presentations with increased odds of COVID-19 mortality. Smoking revealed a protective effect against both the low blood oxygen saturation levels and death in patients with SARS-CoV-2 infection.

The mean age of patients in our study was 53 years old, which was in line with the estimated mean age of COVID-19 patients for outside China (52.5 years old) stated in a systematic review of 10 regions from around the globe. However, it was higher than the findings in China, where the mean age of patients was reported as 46 years old [15]. Compared to a study conducted in Iran early in the epidemic (57.3 years), a slight decrease was noticeable in the mean age of patients with SARS-CoV-2 infection [10]. This age shift could be related partly to the increased number of younger adults returning to workplaces and universities in the past few months. In this study, a greater number of men were infected with SARS-CoV-2 than women (male to female ratio of 1.2). This pattern was observed in most countries and might be attributed to the biological factors which make men generally more susceptible to viral infections [16, 17]. Counts were highest in the public health region of North and East. However, since this public health unit covers a greater proportion of people in the province compared to the other two public health units, this finding was consistent with the expectations. The most common comorbidity reported in COVID-19 patients was hypertension, followed by diabetes, and cardiovascular diseases. The results were aligned with findings from a systematic review of 9249 patients with SARS-CoV-2 infection [18]. The possible explanation for high rates of the abovementioned underlying diseases in patients with COVID-19, is the increased expression of angiotensin-converting enzyme 2 (ACE2) receptors in patients with diabetes and in hypertensive patients treated with ACE2 stimulating medications. ACE2 is believed to have an important role in the SARS-CoV-2 entry into human cells [19]. In accordance with previous studies, the top three common symptoms on admission included cough, difficulty breathing, and fever. Yet considering gastrointestinal presentations, the most common symptoms in this study were nausea and loss of appetite, while diarrhea was the major gastrointestinal complaint reported in literature from outside Iran [9, 15, 20].

Our study revealed that about half of the patients with COVID-19 infection, were suffering from hypoxia at the time of admission, which was higher than the rate reported from China 36%, but lower than the rate in the United States (65%) [21, 22]. However, results must be interpreted with caution given the differences in the age structure and disease severity between the study populations. The ICU admission rate in this study (17.8% of admitted patients) was low in comparison with China (23%), and Italy (21%) [20, 23]. These differences can be justified with the presence of a large proportion of young adults in patients who have been diagnosed with the disease during the epidemic in Tehran. At data cut-off for this study, the overall death rate was 10%, but additional mortalities may happen in those still hospitalised. The overall mortality rate was higher than estimates from Italy (5.6%) or New Zealand (1.5%), but consistent with the estimated death rate in China (11%). The higher death rate obtained in our analysis, could be related to the three consecutive surges of COVID-19 cases during the epidemic. However, it is worth mentioning that the estimated death rates are affected by the number of the tests performed. Higher mortality rates could result when the denominator, the number of the infected individuals, are smaller due to lower testing [24]. Therefore, generalization of the observed mortality rate to the whole population is not recommended.

In the multivariate analysis, patients with the age of 60 and older and male sex were more likely to present with low SpO2 levels on admission, and ultimately die as the result of the disease, which confirms previous findings [6, 11, 20]. Residing in the public health region of North and East was associated with higher risk of low blood oxygen saturation levels in infected patients, whereas, the probability of death was higher for residents of the public health unit of Central and South. Generally north and east areas have higher altitude compared to the rest of the province, and the lower oxygen levels at those higher elevated regions may adversely impact the blood oxygen level in COVID-19 patients and increase their need for supplemental oxygen. Yet, people living in central and south areas are from lower socio-economic status which increases their risk of death when infected with SARS-CoV-2 [25]. Smoking revealed a protective effect against both low blood oxygen saturation level and death in patients with COVID-19. Studies conducted early in the pandemic have reported more severe conditions for smokers with SARS-CoV-2 infection. However, the majority have not considered the important confounders such as age, sex, and existing comorbidities. More recent evidence has shown lower infection rates in smokers and the protective effect of nicotine agent is getting more attention in the literature [26–28]. Having a positive history for opioids dependency was a positive predictor for low SpO2 levels. Yet, its association with COVID-19 related death was insignificant. Similar findings were reported in a study conducted in Spain, however further studies are needed to elucidate the prognosis of patients with COVID-19 who use opioids [28]. Patients with positive history of exposure to SARS-CoV-2 were more likely to develop low blood oxygen levels, but were less likely to develop severe health outcome. Additional work is recommended since studies have shown inconsistent findings in this respect, nevertheless it could be explained by the differences in the load of virus between the two groups [29, 30].

With respect to the comorbidities associated with COVID-19 health outcome, our results revealed that patients with diabetes and cardiovascular diseases have lower odds of developing low SpO2 levels, and that the two underlying diseases were insignificantly associated with COVID-19 death. Hypertension was a positive predictor of low blood oxygen levels, but it was not correlated with death in patients with SARS-CoV-2 infection. These findings do not seem to confirm previous results which have reported these underlying diseases as important contributors of poor prognosis in patients with COVID-19. However, our results seem to be defensible since most studies have only referred to hospitalized patients [28, 31]. Asthma was insignificantly associated with SpO2, but was associated with lower risk of death in COVID-19 infected patients. This is in good agreement with a study performed in United States including both in- and out-patients [32]. However, the presence of other chronic respiratory diseases was associated with both low SpO2 levels and death. Though, there was a negative association between having a history of chronic kidney disease and developing low blood oxygen level, the condition was an independent risk marker for death in patients with COVID-19. Cancer, Chronic liver disease, chronic neurological disorders, and HIV/AIDS infection were insignificantly correlated with low SpO2 levels, nevertheless, they were positively correlated with the mortality due to COVID-19. Immune deficiency disorders other than HIV/AIDS infection and chronic haematological diseases showed no relation with blood oxygen levels or death in SARS-CoV-2 infected patients in our study.

Regarding clinical presentations, fever, difficulty breathing, and impaired consciousness were important risk factors for low blood oxygen saturation level and death. On the other hand, muscle ache, and headache were important protective factors for both conditions. Cough was insignificantly associated with SpO2 levels, but patients with cough had lower risk of death due to the disease. These results corroborate previous findings [6, 28]. Nausea was insignificantly correlated with blood oxygen saturation level, but was negatively associated with mortality. Patients reporting diarrhea had lower odds of developing low blood oxygen levels, but its association with death was not significant. The presence of skin lesions was an adverse predictor for low SpO2 level, but not a significant predictor for death. Our result supports findings from a recent review on this topic, however, the association of cutaneous manifestations and death was reported as significant in the mentioned review article [33].

Finally, our study was relied on secondary analysis of existing data, hence evaluation of factors associated with the health outcomes of patients with COVID-19 were limited to available information. Given the cross-sectional nature of our study drawing conclusions about causal relationships should be done with cautious. As is the issue with most datasets on COVID-19, asymptomatic or mild cases who have not visited the healthcare facilities during the study period were not included in the analyses. Moreover, evaluation of misdiagnosis which was dependent on the sensitivity and specificity of the tests, was not possible with existing data. However, the strength of our study lies in its large multicentre study population which results in more reliable extension of inferences to the target population. Additionally, the registry from where the data was extracted, was the most complete online source of data on COVID-19 patients in the province of Tehran including all patients who have visited the public and private health care facilities in the province and were diagnosed with the disease. Given that the data was collected by trained health care professionals and based on a unified reporting online form, a high-quality data was made available to the researchers allowing real-time processing and analysis of the information.

## Conclusion

The findings of this paper indicate that older age, male sex, suffering from comorbidities included hypertension, cancer, chronic respiratory diseases other than asthma, chronic liver diseases, chronic kidney diseases, chronic neurological disorders, and HIV/AIDS infection are risk markers of poor health outcome in patients with COVID-19. The results might also alert physicians to pay attention to symptoms related with worse prognosis included fever, difficulty breathing, impaired consciousness, and cutaneous manifestations. In addition, findings about the correlates of the severe disease in patients with SARS-CoV-2 infection, aids decision makers to emphasise on vulnerable groups in the public health strategies that aim at preventing the spread of the disease and its mortalities.

## Data Availability

The data that support the findings of this study are available from the Coronavirus Control Operations Headquarter in the province of Tehran, but restrictions apply to the availability of these data, which were used under license for the current study, and so are not publicly available. Data are however available from the corresponding author, Ali-Reza Zali, upon reasonable request and with permission of the Coronavirus Control Operations Headquarter in the province of Tehran.

## References

[1] World Health Organization (2020). Timeline: WHO's COVID-19 response.

[2] World Health Organization (2020). Naming the coronavirus disease (COVID-19) and the virus that causes it.

[3] World Health Organization (2020). Coronavirus disease (COVID-19) pandemic.

[4] Takian A, Raoofi A, Kazempour-Ardebili S (2020). COVID-19 battle during the toughest sanctions against Iran. Lancet.

[5] World Health Organization (2020). The current COVID-19 situation.

[6] Ruan Q, Yang K, Wang W (2020). Clinical predictors of mortality due to COVID-19 based on an analysis of data of 150 patients from Wuhan, China. Intensive Care Med.

[7] Leung K, Wu JT, Liu D (2020). First-wave COVID-19 transmissibility and severity in China outside Hubei after control measures, and second-wave scenario planning: a modelling impact assessment. Lancet.

[8] Karagiannidis C, Mostert C, Hentschker C (2020). Case characteristics, resource use, and outcomes of 10 021 patients with COVID-19 admitted to 920 German hospitals: an observational study. Lancet Respir Med.

[9] Almazeedi S, Al-Youha S, Jamal MH (2020). Characteristics, risk factors and outcomes among the first consecutive 1096 patients diagnosed with COVID-19 in Kuwait. EClinicalMedicine.

[10] Jalili M, Payandemehr P, Saghaei A, Sari HN, Safikhani H, Kolivand P (2020). Characteristics and Mortality of Hospitalized Patients With COVID-19 in Iran: A National Retrospective Cohort Study. Ann Intern Med.

[11] Zali A, Gholamzadeh S, Mohammadi G (2020). Baseline characteristics and associated factors of mortality in COVID-19 Patients; an analysis of 16000 cases in Tehran, Iran. Arch Acad Emerg Med.

[12] Presidency of the I.R.I Plan and Budget Organization. Statistical Center of Iran, https://www.amar.org.ir/english/Statistics-by-Topic/Population-288290-statistical-survey. Accessed 22 Oct 2020.

[13] World Health Organization (2020). WHO COVID-19 Case definition.

[14] Dong Y, Peng CY (2013). Principled missing data methods for researchers. Springerplus.

[15] Li J, Huang DQ, Zou B, Yang H, Hui WZ, Rui F, Yee NTS, Liu C, Nerurkar SN, Kai JCY, Teng MLP, Li X, Zeng H, Borghi JA, Henry L, Cheung R, Nguyen MH (2020). Epidemiology of COVID-19: A systematic review and meta-analysis of clinical characteristics, risk factors, and outcomes. J Med Virol.

[16] Global Health 5050 (2020). The sex, gender and COVID-19 project.

[17] Bwire GM. Coronavirus: why men are more vulnerable to Covid-19 than women? SN Compr Clin Med. 2020:1–3. 2020/08/25. 10.1007/s42399-020-00341-w. 10.1007/s42399-020-00341-w PMC7271824 32838138

[18] Baradaran A, Ebrahimzadeh MH, Baradaran A (2020). Prevalence of comorbidities in COVID-19 patients: a systematic review and meta-analysis. Arch Bone Jt Surg.

[19] Fang L, Karakiulakis G, Roth M (2020). Are patients with hypertension and diabetes mellitus at increased risk for COVID-19 infection?. Lancet Respir Med.

[20] Chen N, Zhou M, Dong X (2020). Epidemiological and clinical characteristics of 99 cases of 2019 novel coronavirus pneumonia in Wuhan, China: a descriptive study. Lancet.

[21] Xie J, Covassin N, Fan Z (2020). Association between hypoxemia and mortality in patients with COVID-19. Mayo Clin Proc.

[22] Bahl A, Van Baalen MN, Ortiz L (2020). Early predictors of in-hospital mortality in patients with COVID-19 in a large American cohort. Intern Emerg Med.

[23] Immovilli P, Morelli N, Antonucci E (2020). COVID-19 mortality and ICU admission: the Italian experience. Crit Care.

[24] Liang LL, Tseng CH, Ho HJ (2020). Covid-19 mortality is negatively associated with test number and government effectiveness. Sci Rep.

[25] Hawkins D (2020). Social determinants of COVID-19 in Massachusetts, United States: an ecological study. J Prev Med Public Health.

[26] Changeux JP, Amoura Z, Rey FA (2020). A nicotinic hypothesis for Covid-19 with preventive and therapeutic implications. C R Biol.

[27] Vardavas CI, Nikitara K (2020). COVID-19 and smoking: A systematic review of the evidence. Tob Induc Dis.

[28] Rodriguez-Molinero A, Galvez-Barron C, Minarro A (2020). Association between COVID-19 prognosis and disease presentation, comorbidities and chronic treatment of hospitalized patients. PLoS One.

[29] Chen X, Wei W, Cao J (2020). Clinical features and short-term outcomes of patients with COVID-19 due to different exposure history. Medicine (Baltimore).

[30] Lian JS, Cai H, Hao SR (2020). Comparison of epidemiological and clinical characteristics of COVID-19 patients with and without Wuhan exposure history in Zhejiang Province, China. J Zhejiang Univ Sci B.

[31] Guo W, Li M, Dong Y, et al. Diabetes is a risk factor for the progression and prognosis of COVID-19. Diabetes Metab Res Rev. 2020:e3319. 2020/04/02. 10.1002/dmrr.3319. 10.1002/dmrr.3319 PMC7228407 32233013

[32] Chhiba KD, Patel GB, Vu THT (2020). Prevalence and characterization of asthma in hospitalized and nonhospitalized patients with COVID-19. J Allergy Clin Immunol.

[33] Matar S, Oules B, Sohier P (2020). Cutaneous manifestations in SARS-CoV-2 infection (COVID-19): a French experience and a systematic review of the literature. J Eur Acad Dermatol Venereol.

